# MaxEnt Modeling of Future Habitat Shifts of *Itea yunnanensis* in China Under Climate Change Scenarios

**DOI:** 10.3390/biology14070899

**Published:** 2025-07-21

**Authors:** Jinxin Zhang, Xiaoju Li, Suhang Li, Qiong Yang, Yuan Li, Yangzhou Xiang, Bin Yao

**Affiliations:** 1 Institute of Ecological Conservation and Restoration, Chinese Academy of Forestry, Beijing 100091, China; 2 Grassland Research Center, National Forestry and Grassland Administration, Beijing 100091, China; 3 School of Geography and Resources, Guizhou Education University, Guiyang 550018, China; 4 Grasslands and Sustainable Farming, Production Systems Unit, Natural Resources Institute Finland, Halolantie 31A, FI-71750 Maaninka, Finland

**Keywords:** *Itea yunnanensis*, climate change, MaxEnt model, habitat suitability prediction, conservation strategies, shared socioeconomic pathways

## Abstract

The distribution of *Itea yunnanensis*, a shrub species in the genus *Itea* of the family *Iteaceae*, is primarily concentrated in the Hengduan Mountains region of China, where it faces severe threats from global climate change. However, systematic research on the species’ distribution patterns, climatic response mechanisms, and future suitable habitat dynamics remains insufficient. This study aims to assess the spatiotemporal evolution and driving mechanisms of *I. yunnanensis*-suitable habitats under current and future climate change scenarios to reveal the migration patterns of its distribution centroid and ecological thresholds, and to enhance the reliability and interpretability of predictions through model optimization. For MaxEnt modeling, we utilized 142 georeferenced occurrence records of *I. yunnanensis* alongside environmental data under current conditions and three future Shared Socioeconomic Pathways (SSPs: SSP1-2.6, SSP2-4.5, SSP5-8.5). Model parameter optimization (Regularization Multiplier, Feature Combination) was performed using the R (v4.2.1) package ‘ENMeval’. The optimized model (RM = 3.0, FC = QHPT) significantly reduced overfitting risk (ΔAICc = 0) and achieved high prediction accuracy (AUC = 0.968). Under current climate conditions, the total area of potential high-suitability habitats for *I. yunnanensis* is approximately 94.88 × 10^4^ km^2^, accounting for 9.88% of China’s land area, with core areas located around the Hengduan Mountains. Under future climate change, the suitable habitats show significant divergence, area fluctuation and contraction under the SSP1-2.6 scenario, and continuous expansion under the SSP5-8.5 scenario. Meanwhile, the species’ distribution centroid exhibits an overall trend of northwestward migration. This study not only provides key spatial decision-making support for the in situ and ex situ conservation of *I. yunnanensis*, but also offers an important methodological reference for the adaptive research on other ecologically vulnerable species facing climate change through its optimized modeling framework.

## Simple Summary

*Itea yunnanensis*, a crucial mountain shrub in China’s Hengduan Mountains for soil stabilization and harboring pharmacologically promising compounds, faces climate change threats. Using calibrated models, we estimate its current range covers about 94.88 × 10^4^ km^2^ (about one-tenth of China), concentrated in the Hengduan region. Future projections diverge: under high emissions, its potential range could expand by 28% by the 2090s, though habitat quality declines. Moderate warming may cause contractions and cyclical shifts. Crucially, the shrub’s core climatic habitat is projected to shift northwestward by up to 310 km as temperatures rise. These findings identify priority zones—including existing reserves and corridors—for proactive conservation of this ecologically and pharmacologically vital species. Our modeling framework can also aid protection strategies for other climate-threatened plants.

## 1. Introduction

*Itea yunnanensis* Franch. (*I. yunnanensis*) is a shrub species belonging to the genus *Itea* of the family *Iteaceae* [1]. The species is predominantly distributed in the Hengduan Mountains region of China, characterized by a narrow distribution range and severely fragmented habitats [2,3]. As an important component of the Hengduan Mountains ecosystem, *I. yunnanensis* plays a key ecological role in maintaining mountain water conservation, soil retention, and microclimate regulation [4,5]. However, the survival of *I. yunnanensis* is currently under severe threat from global climate change: the Hengduan Mountains, as a sensitive region to global warming [6], have experienced continuous contraction of suitable habitats and ecological niche mismatches for high-altitude species due to rising temperatures and intensified seasonal precipitation variability [7]. Meanwhile, the medicinal value of *I. yunnanensis* has increasingly attracted attention. Multiple bioactive compounds, including novel neolignans (e.g., iteanorneoligan A) [8] and 2-arylbenzofuran derivatives [9] have been isolated from its leaves and stem bark, with the latter exhibiting significant anti-hepatocellular carcinoma activity and antioxidant capacity in vitro experiments [10]. High-performance liquid chromatography (HPLC) monitoring has indicated that the key pharmacological component Iteafuranal A reaches its peak accumulation in January (2.63 g/kg), providing a basis for sustainable resource utilization [11]. However, it should be noted that, compared with in-depth chemical and pharmacological studies, the distribution patterns, climatic response mechanisms, and future suitable habitat dynamics of *I. yunnanensis* still lack systematic exploration. This research imbalance not only restricts the formulation of transboundary conservation strategies but may also exacerbate the contradiction between resource development and habitat protection. Therefore, the systematic study of the climate adaptability of *I. yunnanensis* is of great importance and urgency.

Species distribution models (SDMs) have been widely utilized as core tools for analyzing the relationship between species and their environment, playing a crucial role in assessing the impacts of climate change on plant distributions [12,13,14]. Among these models, the MaxEnt model has gained popularity for its adaptability to small sample sizes and high prediction accuracy [15,16,17], making it a commonly used method for predicting the suitable habitats of shrubs and small trees. For instance, in a study on alpine shrublands in the Hengduan Mountains, Zhang et al. [18] employed the MaxEnt model and found, through comprehensive analysis of contribution rates, permutation importance, and Spearman correlation coefficients, that temperature and precipitation are the key drivers influencing the distribution of alpine shrubs, with temperature having a more pronounced effect. In the case of *Taxus wallichiana* Zucc., research using the MaxEnt model, which integrated data from multiple climate models, indicated that with climate warming, the area of its suitable habitat is projected to decrease by 31–55%, and the altitude of its suitable habitat is expected to rise by 390–948 m [19]. A geographical distribution simulation study of *Hippophae rhamnoides* Linn. subsp. sinensis Rousi based on the MaxEnt model revealed that in the hydrothermal co-regulation mechanism, precipitation of wettest month (bio13), mean temperature of coldest quarter (bio11), and isothermality (Bio3) together explained 72.7% of the distribution variation through significant non-monotonic response relationships [20]. However, existing studies still share common limitations: MaxEnt’s regularization inherently mitigates some collinearity effects [21], and default parameters (e.g., RM = 1, FC = LQHP) may inadequately address multicollinearity in high-dimensional or fine-scale analyses. In this study, bioclimatic variables exhibited strong collinearity (|r| > 0.75) at our 2.5-arc-min resolution, necessitating variable screening to avoid overfitting and ensure ecological interpretability.

As a key species in the high-altitude ecosystems of the Hengduan Mountains in China, the narrow niche requirements of *I. yunnanensis* may restrict its potential for climatic adaptation, leading to high vulnerability. However, no studies have yet systematically quantified its adaptive responses to climate change. Therefore, the key issues that this study aims to address include the following: (1) How to improve the reliability and interpretability of species distribution predictions through model parameter optimization. (2) The spatiotemporal evolution characteristics and driving mechanisms of *I. yunnanensis*-suitable habitats under different emission scenarios. (3) The migration patterns of the distribution centroid of *I. yunnanensis* and its ecological adaptation thresholds under climate change. Regarding the first issue, by comparing the model performance with default and optimized parameters, this study will verify the effectiveness of regularization parameter adjustment in reducing overfitting risks and propose a new paradigm for model calibration applicable to high-altitude endemic species. For the second issue, this study will quantify the dynamics of habitat retention, loss, and expansion by constructing presence/absence matrices under modern and future climate scenarios, and determine the optimal climatic combinations for species distribution in conjunction with the response curves of dominant factors. For the third issue, using the ArcGIS centroid analysis toolkit, this study will systematically analyze the migration trajectories of the distribution center of *I. yunnanensis* under multiple scenarios and reveal its spatial response patterns in the context of climatic gradient changes. The results of this study will provide precise spatial decision-making support for the in situ and ex situ conservation of *I. yunnanensis*, as well as methodological references for climate adaptation studies of similar ecologically vulnerable species.

## 2. Materials and Methods

### 2.1. Acquisition and Processing of Distribution Data

**Figure 1:**
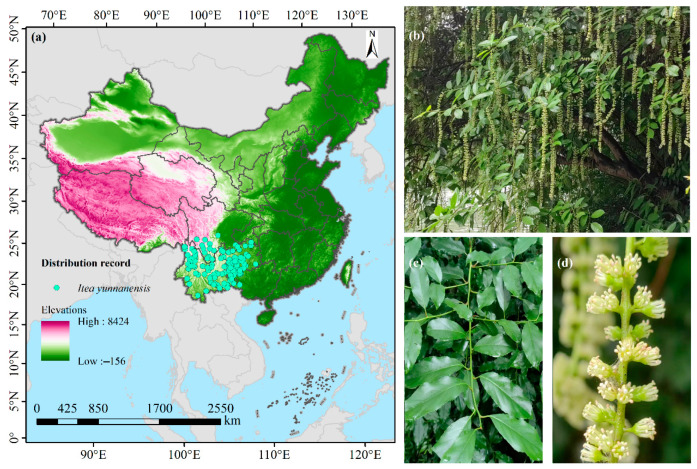
Distribution map of specimens of *I. yunnanensis* in China. (**a**) Distribution points; (**b**) whole tree; (**c**) leaves; (**d**) flowers.

A total of 157 non-duplicated distribution records of *I. yunnanensis* were systematically integrated. These records originated from multiple platforms, including the Chinese Virtual Herbarium (https://www.cvh.ac.cn/, accessed on 8 February 2025); the National Specimen Information Infrastructure (http://www.nsii.org.cn/2017/home.php, accessed on 10 February 2025); the Global Biodiversity Information Facility (https://www.gbif.org/, accessed on 13 February 2025); the China National Knowledge Infrastructure (https://www.cnki.net/, accessed on 14 February 2025); and Google Scholar (https://ac.scmor.com/, accessed on 16 February 2025). To eliminate potential spatial autocorrelation among distribution points and to achieve precise prediction of the distribution pattern of *I. yunnanensis*, the following steps were taken: (1) Grid system construction: A geographic grid of 2.5′ × 2.5′ was precisely constructed using ArcGIS 10.8, providing a standardized and regulated spatial framework for subsequent screening and analysis of distribution points. (2) Distribution point screening and optimization: Within each grid cell, only one distribution point closest to the grid center was retained, following the principles of uniqueness and representativeness. This refined screening process effectively avoided spatial autocorrelation interference among different distribution points, ensuring that the final data points used for analysis were both representative and maximally independent and reliable. After this process, 142 high-quality effective distribution point coordinate data were obtained (Figure 1). (3) Format conversion: According to the data format requirements of MaxEnt 3.4.4, the 142 data points were exported as an Excel 2016 file and then saved as a .csv file, providing standardized and regulated data input for the prediction analysis of the distribution pattern of *I. yunnanensis* using MaxEnt software. The base map of China utilized in the current study was obtained from the Standard Map Service System of the Ministry of Natural Resources of China, with the map approval number being GS (2023)2762 (http://bzdt.ch.mnr.gov.cn/, accessed on 22 December 2023).

### 2.2. Acquisition and Processing of Environmental Data

**Figure 2:**
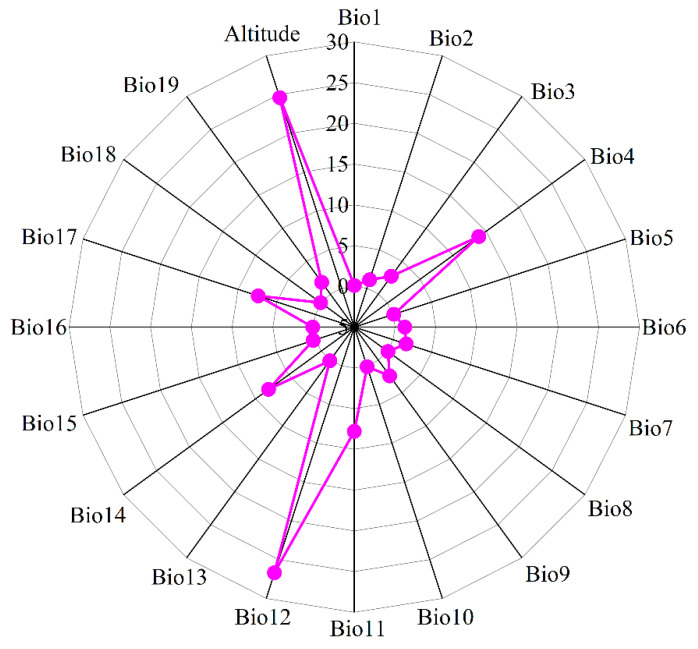
Initial variable importance of 20 ecological factors influencing the habitat distribution of *I. yunnanensis*. Bio1 indicates the annual average temperature; Bio2 refers to the mean diurnal temperature range; Bio3 represents isothermality; Bio4 shows temperature seasonality; Bio5 stands for the maximum temperature of the warmest month; Bio6 is the minimum temperature of the coldest month; Bio7 means the temperature annual range; Bio8 reflects the mean temperature of the wettest quarter; Bio9 denotes the mean temperature of the driest quarter; Bio10 signifies the mean temperature of the warmest quarter; Bio11 corresponds to the mean temperature of the coldest quarter; Bio12 gives the annual precipitation; Bio13 presents the precipitation of the wettest month; Bio14 shows the precipitation of the driest month; Bio15 represents precipitation seasonality; Bio16 stands for the precipitation of the wettest quarter; Bio17 is the precipitation of the driest quarter; Bio18 indicates the precipitation of the warmest quarter; Bio19 signifies the precipitation of the coldest quarter.

**Figure 3:**
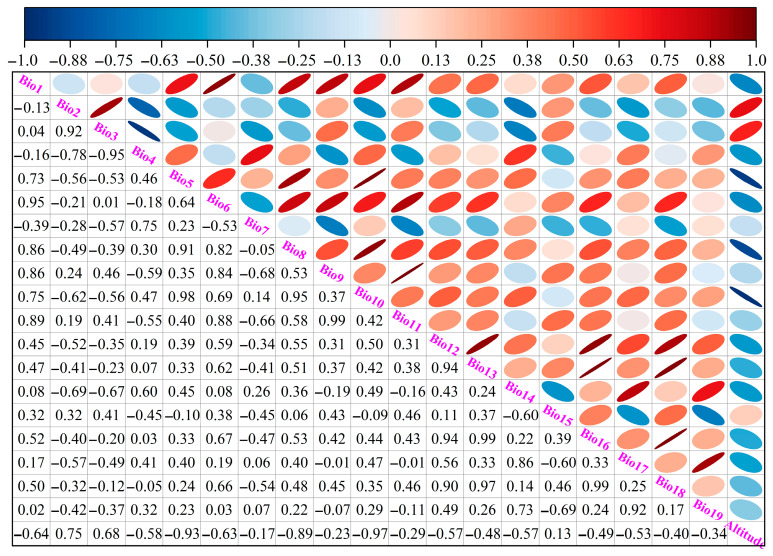
The Spearman correlation analysis involving 20 environmental factors in this study. Ellipse minor-axis lengths shrink as factor correlation coefficients grow in magnitude. Intensifying blue signifies steeper negative correlations, whereas deepening red denotes stronger positive ones.

The environmental data utilized in this study were sourced from the WorldClim database, a global climate data center that provides 19 bioclimatic variables (bio1-bio19) for both contemporary and future climate scenarios, covering key climatic elements such as temperature, precipitation, and seasonal variations. Additionally, the elevation data were also obtained from WorldClim. The spatial resolution of the 20 environmental variables (Figure 2) was uniformly set at 2.5′ × 2.5′. Future climate data encompassed three time periods—the 2050s (2041–2060), the 2070s (2061–2070), and the 2090s (2081–2100)—which were selected to effectively capture the potential impacts of climate change on species distribution at different stages. Regarding the choice of climate models, the BCC-CSM2-MR model was selected based on its high accuracy and reliability in simulating climate conditions over China as part of the Coupled Model Intercomparison Project Phase 6 (CMIP6). This study focused on three Shared Socioeconomic Pathways (SSPs) under the BCC-CSM2-MR model: SSP1-2.6 (low emission scenario, with an estimated global temperature increase of approximately 1.8 °C by 2100), SSP2-4.5 (medium emission scenario, with an estimated global temperature increase of approximately 2.7 °C by 2100), and SSP5-8.5 (high emission scenario, with an estimated global temperature increase of approximately 4.4 °C by 2100) [22]. In the data processing phase, Spearman correlation analysis was conducted on the 20 environmental variables using Origin 2025b software (Figure 3), and the contribution rates of the variables calculated by MaxEnt 3.4.4 (http://biodiversityinformatics.amnh.org/open_source/maxent/, accessed on 21 February 2025) software were comprehensively assessed. If the absolute value of the correlation coefficient between two environmental variables exceeded 0.75, the variable with the lower contribution rate was removed. This approach was adopted to minimize data redundancy and enhance the efficiency and interpretability of the model. Finally, six environmental factors, involving temperature seasonality (Bio4), mean temperature of coldest quarter (Bio11), annual precipitation (Bio12), precipitation of driest month (Bio14), precipitation seasonality (Bio15), and altitude, were retained to predict the geographic distribution of *I. yunnanensis* under different climate conditions.

### 2.3. MaxEnt Model Optimization and Modeling

#### 2.3.1. Optimization of MaxEnt Model

Generally, the probability predictions of uncalibrated models may not align with the true distribution of species [23]. Utilizing uncalibrated models to predict the potential distribution of *I. yunnanensis* could lead to overestimation or underestimation of suitable habitat ranges due to probability distortions, thereby invalidating the scientific basis for delineating conservation priority areas. Therefore, optimizing the parameters of the MaxEnt model is crucial for accurately assessing the impacts of climate change on its future distribution. In this study, the MaxEnt model was optimized using the R (v4.2.1) package ‘ENMeval’ [24] by adjusting two key parameters—Regularization Multiplier (RM) and Feature Combination (FC). The MaxEnt model comprises five types of features: Linear (L), Quadratic (Q), Product (P), Threshold (T), and Hinge (H). Nine feature combinations (FC) were set in this study, namely L, H, LQ, HPT, LQH, QHPP, QHPT, and LQHPT, along with eight Regularization Multiplier (RM) values ranging from 0.5 to 4.0 (with a step size of 0.5) [25]. To evaluate the fit and complexity of different parameter combinations, delta.AICc and OR10 were selected as evaluation indicators [21], and the optimal parameter combination was chosen based on these criteria for modeling.

#### 2.3.2. MaxEnt Model Parameter Setting

The potential suitable habitats of *I. yunnanensis* were predicted in this study based on the MaxEnt model optimized with parameters (RM = 3.0, FC = QHPT). A total of 142 valid distribution points were used, which were divided into a training set (106 points, 75%) and a testing set (36 points, 25%) through random stratified sampling. Cross-validation was set up with 10 replicates, and the maximum number of background points was limited to 10,000 to control overfitting. The final output was generated as a Logistic probability .asc raster file to meet the needs of spatial analysis.

#### 2.3.3. Evaluation of MaxEnt Model Results

The Receiver Operating Characteristic Curve (ROC) and its Area Under the Curve (AUC) [21] were employed in this study as the quantitative evaluation criteria for the MaxEnt model to establish a systematic model performance assessment framework. The AUC value ranges from 0 to 1 [26,27], and its biological significance is determined based on threshold levels as follows: an AUC value of 0.5–0.6 indicates that the model’s predictive performance is not significantly different from random distribution (*p* > 0.05), and is deemed an ineffective model; an AUC value of 0.6–0.7 signifies that the model only has basic discriminative ability, with insufficient reliability in prediction results; an AUC value of 0.7–0.8 reflects that the model can effectively distinguish between suitable and unsuitable habitats for *I. yunnanensis*, meeting the basic requirements of niche modeling; an AUC value of 0.8–0.9 indicates that the model has high-precision predictive capability with significant spatial fit; and an AUC value of 0.9–1.0 suggests that the model nearly perfectly reconstructs the actual distribution pattern of the species [28].

### 2.4. Delineation of Suitable Habitats for I. yunnanensis

The prediction of suitable habitats for *I. yunnanensis* was ensured to be repeatable and scientific through a systematic modeling process in this study. Initially, an ensemble modeling strategy was employed, integrating the mean results of 10 independent MaxEnt model runs to create a spatial decision surface, effectively reducing the interference of random errors on prediction stability. The original ASC format output was converted into TIF raster data using ArcGIS 10.8 software. The Jenks Natural Breaks Classification algorithm in the “Reclassify” tool was utilized to delineate the suitability levels for *I. yunnanensis* [29]: areas with a suitability probability of less than 0.1 were defined as unsuitable, representing regions of complete niche mismatch; areas with probabilities of 0.1–0.3 were categorized as low suitability, reflecting potential expansion zones under stressful environments; areas with probabilities of 0.3–0.5 were classified as medium suitability, corresponding to suboptimal habitats with partial niche matches; and areas with probabilities of 0.5–1 were designated as high suitability, indicating priority conservation areas where the niche of *I. yunnanensis* is highly matched.

### 2.5. Spatial Pattern Changes in Suitable Habitats for I. yunnanensis

The distribution of suitable habitats for *I. yunnanensis* was predicted based on the optimized MaxEnt model in this study, with areas having a logistic value ≥ 0.1 defined as suitable habitats and those with a logistic value < 0.1 defined as unsuitable habitats. By constructing presence/absence (1, 0) matrices under current and future climate conditions and based on the modern potential suitable habitat area of *I. yunnanensis*, future area changes were analyzed. The matrix value changes have clear ecological significance: 0→1 indicates areas of gain, 1→0 indicates areas of loss, and 1→1 indicates areas of retention. The matrix values were converted to attribute values using ArcGIS 10.8 software to create maps of spatial distribution changes in suitable habitats for *I. yunnanensis*.

### 2.6. Centroid Migration of Suitable Habitats for I. yunnanensis

The centroid changes in suitable habitat areas for *I. yunnanensis* under different time nodes and climate scenarios were systematically analyzed in this study using the ArcToolbox in ArcGIS 10.8 software. The specific steps are as follows: (1) The .tif data were reclassified using the “reclassify” tool. (2) The raster data were converted into distribution points through the “raster to point” tool. (3) The distribution center was extracted using the “mean center” tool. (4) The above steps were repeated to obtain the suitable distribution centers under three climate scenarios. (5) The suitable center point data under all climate scenarios were integrated into a single vector dataset using the “point merge” tool. (6) The point data from different years were integrated and connected using the “points to line” tool to form an intuitive migration arrow diagram.

## 3. Results

### 3.1. Model Optimization and Accuracy Assessment

**Figure 4:**
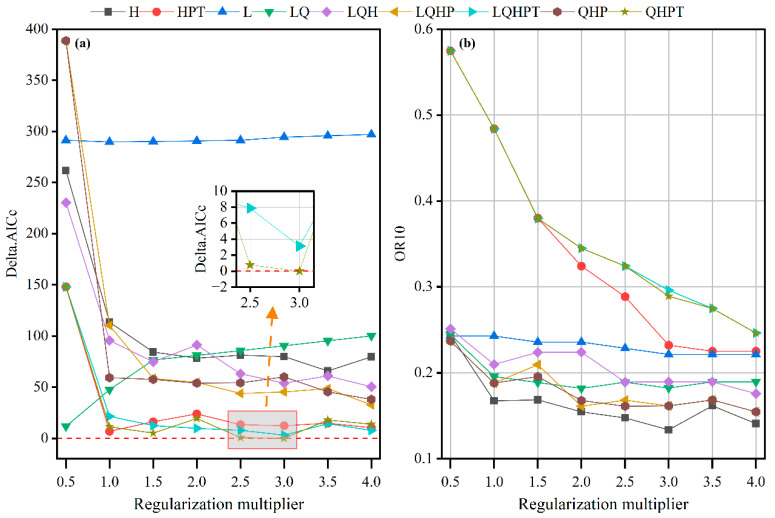
ENMeval-based model evaluation metrics for *I. yunnanensis*. (**a**) Delta.AICc and (**b**) OR10 derived from ENMeval. Feature categories in legend: L (Linear), Q (Quadratic), H (Hinge), P (Product), T (Threshold). The short horizontal red dashed line indicates the zero reference.

The optimization effect of MaxEnt modeling parameters was evaluated using Delta.AICc and OR10 in this study [30]. When the initial default parameter configuration (RM = 1, FC = LQHP) was used, the model’s Delta.AICc value reached 110.3069 (Figure 4a), significantly exceeding the overfitting threshold of Delta.AICc > 2. This high Delta.AICc value indicates that the default parameters may lead to overfitting, where the model fits the training data too closely and performs poorly on new, unseen data. To address this issue and improve the model’s generalizability, parameter optimization is necessary. Using the ENMeval package to process the distribution point coordinates and data of six dominant environmental factors of *I. yunnanensis*, we systematically evaluated different combinations of Regularization Multiplier (RM) and Feature Combination (FC). Two effective parameter combinations with Delta.AICc < 2 were obtained: RM = 2.5/FC = QHPT (Delta.AICc = 0.7737) and RM = 3.0/FC = QHPT (Delta.AICc = 0). Both combinations met the model selection criteria of the AICc information criterion, indicating a good trade-off between model fit and complexity.

**Figure 5:**
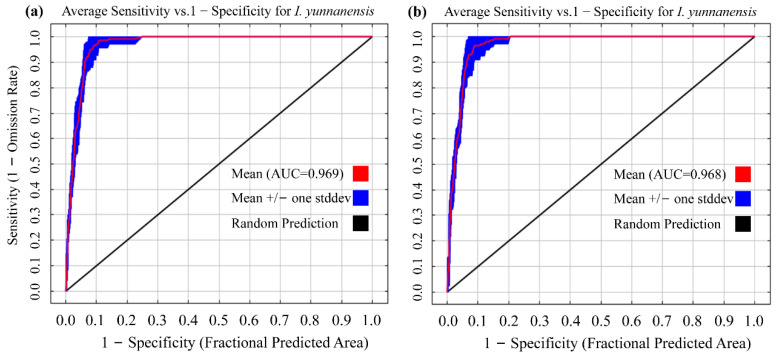
Accuracy evaluation of the MaxEnt model for *I. yunnanensis* via ROC. (**a**) AUC from default parameters; (**b**) AUC from optimized parameters.

Further validation through the OR10 indicator revealed that the OR10 value for the RM = 2.5 combination was 0.3240, 11.7% higher than that for the RM = 3.0 combination (OR10 = 0.2891). This suggests that the RM = 3.0 combination had superior predictive conservatism, meaning it is less likely to overfit the training data and provides more reliable predictions on new data. Based on the principle of parsimony [31], which advocates for the simplest model that adequately explains the data, the combination of RM = 3.0/FC = QHPT was ultimately selected as the optimal parameter configuration. This configuration not only maintained the theoretically optimal value of Delta.AICc (Δ = 0) but also achieved the best balance between prediction accuracy and complexity. The slight decrease in AUC (by 0.01%) when using the optimized parameters compared to the default parameters is a trade-off for reducing the risk of overfitting. This approach aligns with the principles of regularization in statistical modeling, where the goal is to penalize overly complex models to improve their generalization ability. The necessity of parameter tuning in MaxEnt modeling is well-documented in the literature. For instance, Phillips and Dudík [32] emphasized that the performance of MaxEnt is significantly influenced by its parameters, and tuning these parameters can substantially improve model accuracy. They also highlighted that default parameter settings may not always be optimal for every dataset, especially when dealing with small sample sizes or highly variable environmental data. Our study followed a similar approach by empirically determining the optimal parameter settings that minimize overfitting risk while maintaining high prediction accuracy (Figure 5).

### 3.2. The Main Environmental Factors Influencing the Distribution of I. yunnanensis

**Figure 6:**
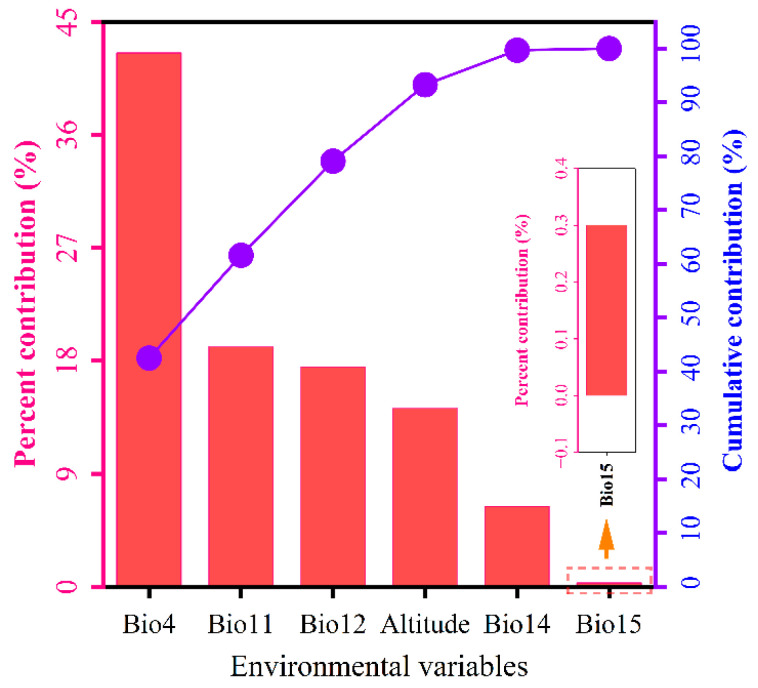
Relative importance of six environmental factors for *I. yunnanensis* via Jackknife Testing. The purple polyline represents the cumulative contribution rate.

**Figure 7:**
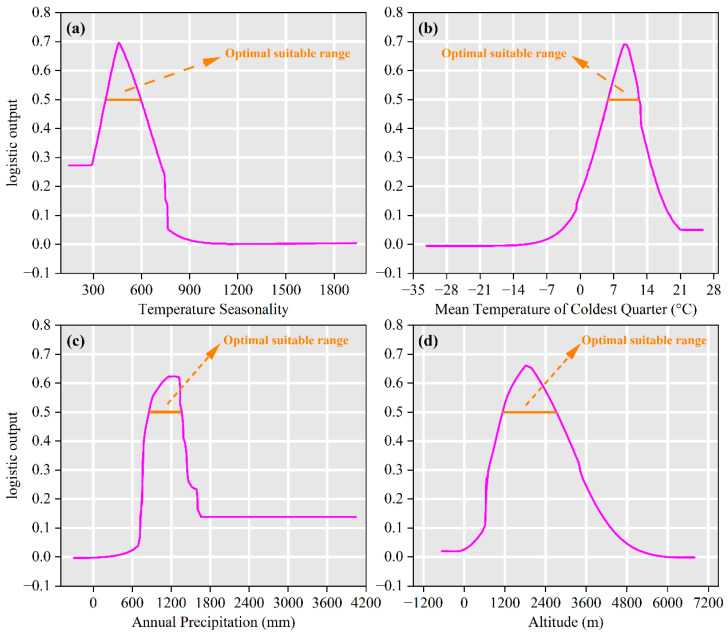
Response curves for key environmental predictors in the species distribution model for *I. yunnanensis*. (**a**) Temperature seasonality (Bio4); (**b**) mean temperature of coldest quarter (Bio11); (**c**) annual precipitation (Bio12); (**d**) altitude.

Environmental factor contributions identified temperature seasonality (Bio4, 42.5%), mean temperature of coldest quarter (Bio11, 19.1%), annual precipitation (Bio12, 17.5%), elevation (14.2%), precipitation of driest month (Bio14, 6.4%), and precipitation seasonality (Bio15, 0.3%) as the primary climatic drivers governing *I. yunnanensis* distribution (Figure 6). Based on the cumulative contribution threshold exceeding 85% [29], Bio4, Bio11, Bio12, and Elevation (cumulative 93.3%) were recognized as dominant determinants of contemporary distribution patterns. Response curve analysis revealed unimodal responses: distribution probability peaked at 460.43 °C for Bio4 (301.21–767.62 °C), 9.48 °C for Bio11 (−6.85–21.33 °C), 1150.60 mm for Bio12 (305.31–1657.02 mm), and 1810.51 m for Elevation (0–5826.69 m), with all exhibiting initial increases followed by declines (Figure 7). According to Yang et al. [33], the environmental ranges corresponding to a species distribution probability ≥ 0.5 are considered optimal suitability thresholds. Therefore, the most suitable habitat for *I. yunnanensis* was defined as regions where Bio4 ranged from 376.14 to 597.12 °C, Bio11 from 5.79 to 12.32 °C, Bio12 from 854.86 to 1359.86 mm, and elevation from 1138.36 to 2704.42 m.

### 3.3. Current Potential Suitable Habitats of I. yunnanensis

**Figure 8:**
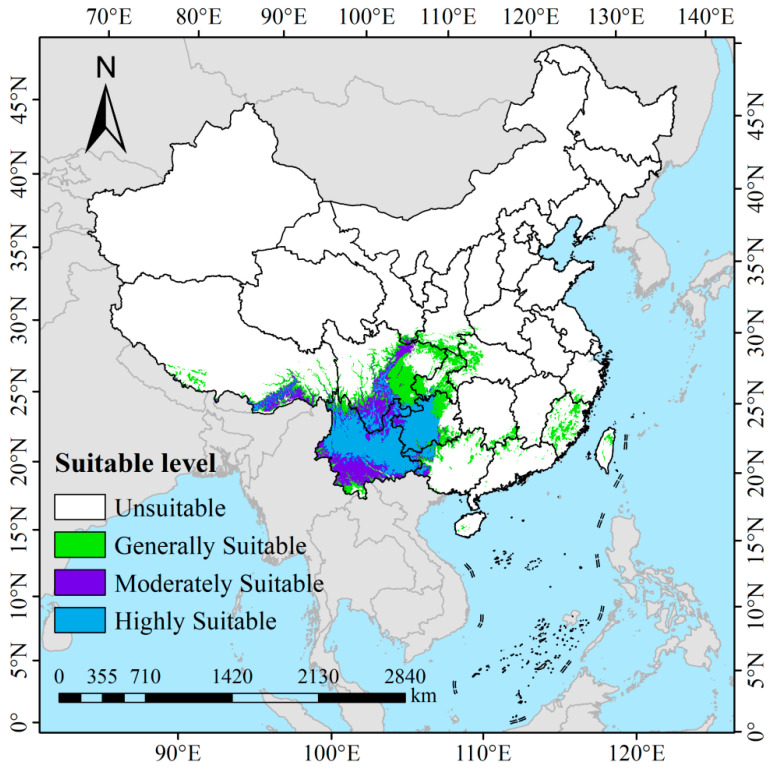
Present habitat suitability projections for *I. yunnanensis* in China.

Under current climatic conditions, the total potential suitable habitat area for *I. yunnanensis* was estimated at 94.88 × 10^4^ km^2^, accounting for 9.88% of China’s land area (Figure 8). The distribution was characterized as follows: (1) the low-suitability zone, covering approximately 38.08 × 10^4^ km^2^ (3.97% of China’s land area), was primarily located in northern Taiwan, central Fujian, southern Hunan, northern Guizhou, eastern Sichuan, central Chongqing, western Hubei, and parts of southern Yunnan; (2) the medium-suitability zone, encompassing about 21.35 × 10^4^ km^2^ (2.22% of China’s land area), was mainly distributed in southeastern Tibet, central–southern Sichuan, southern Yunnan, and western Guizhou; (3) the high-suitability zone, spanning roughly 35.45 × 10^4^ km^2^ (3.69% of China’s land area), was predominantly concentrated in the surrounding areas of the Hengduan Mountains, including most regions of southern Sichuan, northern Yunnan, and western Guizhou.

### 3.4. Potential Suitable Habitats of I. yunnanensis Under Future Climate Change

**Figure 9:**
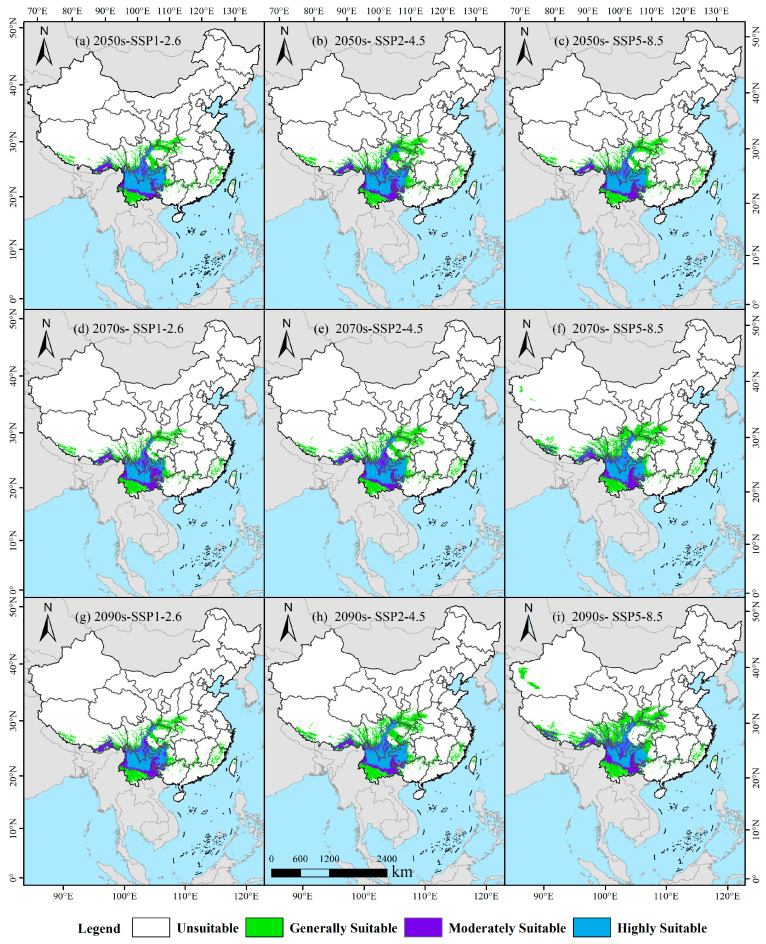
Projected suitable habitats for *I. yunnanensis* across three future periods (2041–2060, 2061–2080, 2081–2100) under varied climate scenarios.

Under the SSP1-2.6 climate scenario, distinct temporal variations were observed in the suitable habitat area of *I. yunnanensis*. During 2041–2060, the total suitable area was estimated at 101.14 × 10^4^ km^2^ (Table 1, Figure 9a), comprising 30.41 × 10^4^ km^2^ of high-suitability zones (primarily distributed in western Guizhou, southern Sichuan, and northern Yunnan), 20.55 × 10^4^ km^2^ of medium-suitability zones, and 50.18 × 10^4^ km^2^ of low-suitability zones. By 2061–2080, a marginal reduction to 93.73 × 10^4^ km^2^ was recorded, with high-suitability areas decreasing to 21.19 × 10^4^ km^2^ (showing northwestward expansion in Guizhou), while medium- and low-suitability zones increased to 24.45 × 10^4^ km^2^ and 48.08 × 10^4^ km^2^ respectively. The period 2081–2100 witnessed a recovery to 101.24 × 10^4^ km^2^, featuring 25.53 × 10^4^ km^2^ of high-suitability areas that became reconcentrated in the original core regions. The SSP2-4.5 scenario revealed an initial increase followed by stabilization in habitat suitability. The total area expanded from 113.99 × 10^4^ km^2^ (2041–2060; 28.20 × 10^4^ km^2^ high-suitability) to 118.18 × 10^4^ km^2^ (2081–2100; 30.69 × 10^4^ km^2^ high-suitability), with notable northward expansion of high-suitability zones into central–southern Sichuan during intermediate periods. Medium-suitability areas demonstrated progressive contraction from 25.43 × 10^4^ km^2^ to 22.62 × 10^4^ km^2^, whereas low-suitability zones exhibited consistent growth from 60.35 × 10^4^ km^2^ to 64.88 × 10^4^ km^2^. Most significantly, under the high-emission SSP5-8.5 scenario, a continuous expansion trend was identified. The total suitable area grew progressively from 105.85 × 10^4^ km^2^ (2041–2060) to 135.31 × 10^4^ km^2^ (2081–2100), representing a 27.8% increase. This expansion was predominantly driven by low-suitability zones, which surged from 55.98 × 10^4^ km^2^ to 80.02 × 10^4^ km^2^ (+43.0%), while high-suitability areas showed modest decline from 28.43 × 10^4^ km^2^ to 25.58 × 10^4^ km^2^. The core high-suitability distribution remained remarkably stable, persistently concentrated in western Guizhou, central–southern Sichuan, and northern Yunnan throughout all periods. These patterns suggest that climate change intensification may facilitate substantial range expansion for *I. yunnanensis*, particularly in marginal habitats under high-emission scenarios.

### 3.5. Habitat Suitability Dynamics Under Future Climate Scenarios

**Figure 10:**
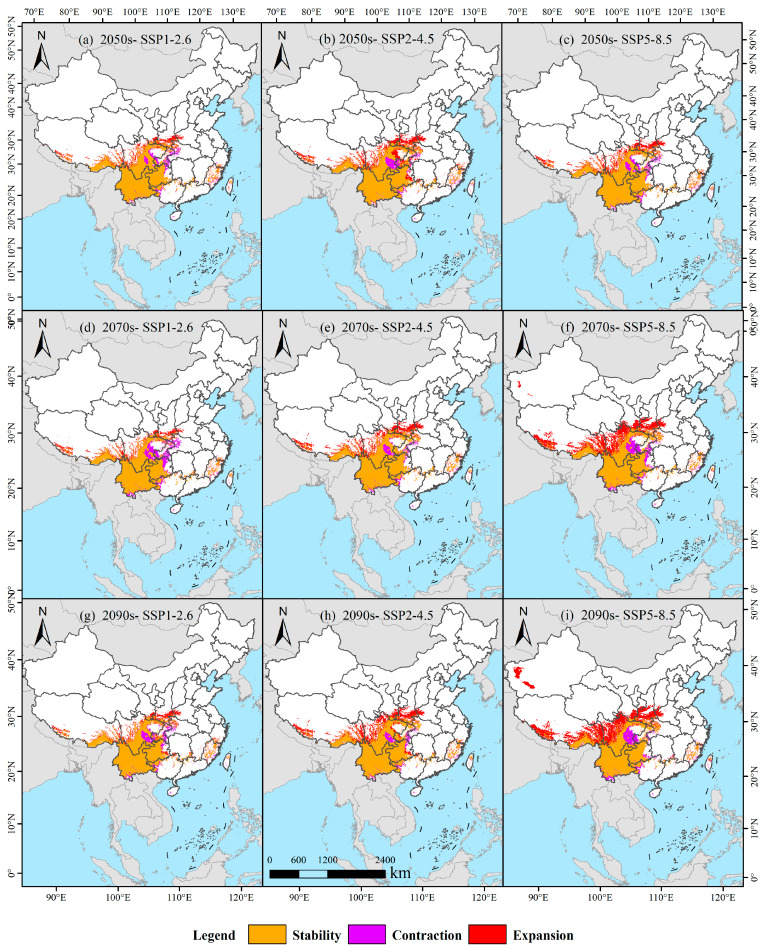
Habitat suitability dynamics of *I. yunnanensis* under present climate conditions.

Under SSP1-2.6, *I. yunnanensis* habitat dynamics exhibited phased shifts (Table 2): relative to current conditions, retained suitability areas were recorded at 104.85 × 10^4^ km^2^ (76.88%), lost areas at 11.81 × 10^4^ km^2^ (8.66%), and expanded areas at 19.73 × 10^4^ km^2^ (14.47%) during 2041–2060 (Figure 10a). By 2061–2080, retained area declined to 97.35 × 10^4^ km^2^ (72.25%) with increased loss (19.31 × 10^4^ km^2^, 14.33%) and reduced expansion (18.09 × 10^4^ km^2^, 13.43%) (Figure 10d). In 2081–2100, recovery was observed in retained area (103.08 × 10^4^ km^2^, 74.53%), while loss decreased (13.57 × 10^4^ km^2^, 9.81%) and expansion increased (21.66 × 10^4^ km^2^, 15.66%) (Figure 10g). Under SSP2-4.5, stable trends were demonstrated: initial retention (105.84 × 10^4^ km^2^, 69.95%), loss (10.80 × 10^4^ km^2^, 7.14%), and expansion (34.67 × 10^4^ km^2^, 22.91%) during 2041–2060 (Figure 10b). Retention slightly increased to 106.96 × 10^4^ km^2^ (73.06%) by 2061–2080 with reduced loss (9.69 × 10^4^ km^2^, 6.62%) and expansion (29.74 × 10^4^ km^2^, 20.32%) (Figure 10e). By 2081–2100, retention further rose (107.85 × 10^4^ km^2^, 69.74%), loss decreased (8.78 × 10^4^ km^2^, 5.68%), and expansion surged (38.01 × 10^4^ km^2^, 24.58%) (Figure 10h). Under SSP5-8.5, high-emission characteristics were manifested: early-stage retention (104.61 × 10^4^ km^2^, 73.36%), loss (12.04 × 10^4^ km^2^, 8.45%), and expansion (25.94 × 10^4^ km^2^, 18.19%) during 2041–2060 (Figure 10c). By 2061–2080, retention sharply declined to 100.82 × 10^4^ km^2^ (59.35%) with increased loss (15.83 × 10^4^ km^2^, 9.32%) and expanded area (53.24 × 10^4^ km^2^, 31.34%) (Figure 10f). By 2081–2100, retention decreased further (100.62 × 10^4^ km^2^, 54.98%), loss slightly increased (16.03 × 10^4^ km^2^, 8.76%), and expansion substantially rose (66.38 × 10^4^ km^2^, 36.27%) (Figure 10i).

### 3.6. Climate-Driven Centroid Migration of I. yunnanensis Across Emission Scenarios

**Figure 11:**
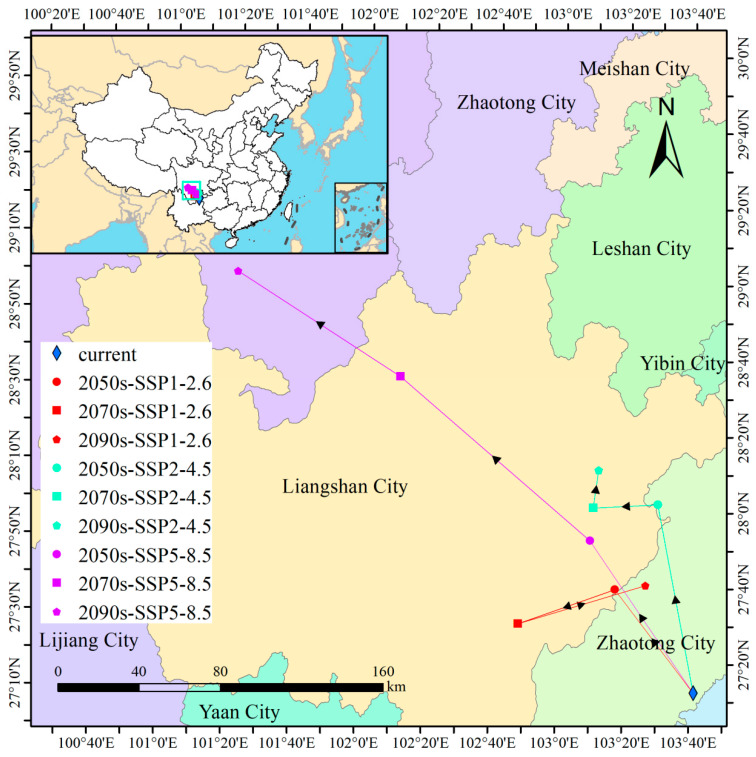
Distribution centroid of *I. yunnanensis* in China under future climate conditions. The black arrow indicates the direction of migration.

Multisimulation analyses project an overall northwestward migration of *I. yunnanensis*’ distribution centroid driven by climate-change-induced thermal suitability gradients (Figure 11). The current centroid, serving as the spatial baseline for trajectory assessment, is located at Buga Township, Zhaoyang District, Yunnan Province (103°41′ E, 27°12′ N). Under SSP1-2.6 (low radiative forcing), migration exhibited nonlinear dynamics: a 63.97 km northwest displacement to Mayizu Township, Sichuan Province (2041–2060; 3.20 km/yr) was followed by a 50.74 km southwest reversal to Laguo Township, Butuo County (2061–2080), and culminated in a 65.76 km northeast shift to Daxing Town, Yongshan County (2081–2100), yielding a net 58.43 km northwest displacement. This oscillatory pattern indicates niche stability maintenance through local adaptation. Under SSP2-4.5 (moderate forcing), progressive northwest migration was observed: a 93.86 km displacement to Shuizhu Township, Zhaotong (2041–2060; 4.69 km/yr; +0.83° latitude) preceded shorter southwest (32.08 km) and northeast (18.64 km) movements (2061–2100), resulting in a net 111.38 km displacement with +0.99° latitude, confirming thermal optimization through directional migration. Under SSP5-8.5 (high emissions), explosive northwest migration occurred: an initial 90.46 km shift to Bingdi Town, Jinyang County (2041–2060; 4.52 km/yr; +642 m elevation) accelerated to 123.63 km northwest to Yihai Town, Mianning County (2061–2080; 6.18 km/yr; +0.72°/decade latitude), concluding with a 95.64 km displacement to Sanyanlong Township, Jiulong County (2081–2100). This formed a total 309.73 km migration corridor with +1483 m net elevation gain. The exponential flux increases and consistent northwest vector under high emissions demonstrate climate-driven niche displacement exceeding local adaptation thresholds, necessitating distribution reconstruction through long-distance migration.

## 4. Discussion

### 4.1. MaxEnt Model Parameter Optimization and Performance

Systematic optimization of MaxEnt model parameters (Regularization Multiplier RM = 3.0, Feature Combination FC = QHPT) was implemented, achieving significant reduction in overfitting risk (ΔAICc = 0) while maintaining high predictive accuracy (AUC = 0.968). This outcome demonstrates that parameter optimization constitutes not merely a technical adjustment but also is a critical step for deciphering ecological mechanisms in niche modeling of high-elevation endemics [34]. Specifically, elevation of the Regularization Multiplier (from default 1 to 3) effectively suppressed overfitting to noise data by constraining model complexity, such as through the reduction of weights for higher-order polynomial features [35]. Concurrently, feature combination optimization (QHPT) was executed through elimination of linear features and incorporation of threshold features, enabling more precise capture of nonlinear environmental responses in *I. yunnanensis* [36]. For instance, its response curve to mean temperature of coldest quarter (Bio11) exhibited a pronounced threshold effect (peak at 9.48 °C), a niche boundary inadequately captured by default linear or quadratic features. Further analysis revealed an 11.7% reduction in the OR10 metric (0.2891 vs. default 0.3240) in the optimized model, indicating substantially enhanced prediction conservatism [37]. These results underscore the heightened sensitivity of narrowly distributed species to model parameters, where default settings may cause the over-smoothing of responses to climatic extremes [38].

Consistent with the findings of Zhang et al. [18] on *Rhododendron lapponicum* (L.) Wahlenb. in the Hengduan Mountains, this study demonstrates that parameter optimization enhances the robustness of models for endemic species. However, it contrasts sharply with the research by Zhang et al. [39] on Prunus pedunculata Pall., which, using default parameters (RM = 1, FC = LQPH), predicted a reduction of approximately 7.1 × 10^4^ km^2^ in suitable habitat under high-emission scenarios (2050s-SSP5-8.5). In contrast, optimized model parameters projected an increase of about 2.6 × 10^4^ km^2^ in suitable habitat for the same species and emission scenario [40], suggesting that unoptimized models may overestimate the threats of climate change to species distributions. This study integrated multisource distribution data, including herbarium records, the literature, and field surveys, and employed gridded screening at a 2.5′ resolution to significantly reduce spatial autocorrelation and enhance data quality [41,42]. Early studies often relied on single databases [43], which may introduce model bias due to sampling bias, such as over-sampling in urban vicinities [44]. These findings offer methodological insights for future research: for distribution modeling of endemic species, parameter optimization and multisource data cleaning strategies should be prioritized to balance ecological interpretability and predictive reliability.

The projected distribution shifts reveal critical ecological thresholds governing *I. yunnanensis* persistence. The 12.3% reduction in peak suitability per 100 °C increase in temperature seasonality (Bio4) reflects the species’ limited phenotypic plasticity to thermal variability, consistent with its evolutionary history in climatically stable cloud forests. The precipitation threshold of 855 mm (Bio12) represents a physiological tipping point where stomatal regulation fails to maintain positive carbon balance, explaining the sharp eastern range limit. The 309.73 km northwestward centroid migration under SSP5-8.5 follows elevation-dependent warming patterns, with current optimal habitats (2000–2700 m) experiencing temperatures exceeding the species’ thermal tolerance envelope (>15 °C mean annual temperature). This forces populations to track suitable microclimates along the Hengduan Mountain corridors, utilizing deep valleys as thermal refugia where cold-air pooling maintains viable temperature regimes.

### 4.2. Key Environmental Variable Interactions and Ecological Threshold

The distribution pattern of *I. yunnanensis* was primarily driven by temperature seasonality (Bio4, 42.5%), mean temperature of coldest quarter (Bio11, 19.1%), and annual precipitation (Bio12, 17.5%), collectively accounting for 79.1% contribution. These bioclimatic thresholds align with *I. yunnanensis*’s functional traits. The optimum Bio11 of 9.48 °C matches its documented chilling requirement for synchronized budburst after winter dormancy [8]. Its shallow, fine-root system explains the sharp decline in suitability when Bio12 < 855 mm, because dry-season soil-moisture deficits cannot be buffered by deep water uptake [45]. Conversely, the peak at 1150 mm coincides with the cloud-forest zone where persistent fog reduces transpirational demand and supports year-round photosynthetic activity [46]. High standard deviation of temperature seasonality (Bio4 ≈ 460 °C) is tolerable because sclerophyllous leaves limit photoinhibition during large diurnal temperature swings typical of high-elevation valleys [47].

Consensus with He et al. [20] regarding *Hippophae rhamnoides* L. was observed, where hydrothermal synergy (Bio12 and Bio11) was similarly identified as the dominant factor. However, significant divergence from Guo et al. [48] on subtropical deciduous broad-leaved forests was noted—isothermality (Bio3) contributed 23.52% in the latter, whereas *I. yunnanensis* exhibited greater sensitivity to temperature seasonality than annual temperature stability. This discrepancy is attributed to life-history strategy and habitat heterogeneity interactions: as an evergreen shrub, its sclerophyllous leaves and C3 photosynthetic pathway require stable seasonal temperature differences to drive phenological rhythms [47], while subtropical trees (e.g., *Quercus* spp.) rely more on annual temperature stability for sustained photosynthetic activity [49]. Additionally, topographic fragmentation in the Hengduan Mountains intensifies environmental heterogeneity, where micro-scale climatic variations (<10 km^2^, e.g., valley-ridge temperature differentials) may mask large-scale precipitation seasonality (Bio15) effects [50,51]. This explains the minimal contribution of Bio15 (0.3%) in the present study, contrasting with its 34.9% contribution in Wang et al. [52] for potential distribution modeling of tree species in northern China’s arid regions.

### 4.3. Climate-Driven Habitat Suitability Dynamics: Path Dependency and Migration Constraints

Significant path dependency was exhibited in *I. yunnanensis* habitat suitability changes across emission scenarios. Specifically, under SSP1-2.6 (low emissions), fluctuating contraction in suitable area was observed (74.5% retention by the 2090s), with centroid migration characterized by “NW-SW-NE” oscillation (net displacement: 58.43 km). Conversely, under SSP5-8.5 (high emissions), substantial expansion of low-suitability areas was recorded (36.3% increase by 2090s), but accompanied by accelerated loss of high-suitability habitats (25.58 × 10^4^ km^2^), alongside continuous northwestward centroid migration (309.73 km) with 1483 m elevation gain. This phenomenon was attributed to spatial heterogeneity in climate variability: rapid warming under SSP5-8.5 was linked to the disappearance of core habitats (e.g., 2000–2700 m cloud forests) through climatic “squeeze effects” [53,54], while low-suitability expansion reflected niche generalization capacity (e.g., extended flowering phenology via phenotypic plasticity to adapt to broader temperature fluctuations) [55]. Simultaneously, the northwestward migration preference was closely associated with the N-S orientation of the Hengduan Mountains’ topographic corridors—longitudinal valleys were identified as facilitating migration pathways [56], while the eastern plains were formed into migration barriers by anthropogenic disturbances (e.g., agricultural expansion and urbanization).

Multi-scenario simulations converge on a consistent northwestward shift in the centroid of *I. yunnanensis*’ range, propelled by climate-driven gradients in thermal suitability (Figure 11). Specifically, rising temperatures (especially in the coldest quarter, Bio11) force this heat-sensitive species toward higher latitudes to track optimal thermal niches, while reduced precipitation in core habitats (e.g., Bio12 < 855 mm) drives migration along humidity gradients preserved in mountainous corridors. Consensus with Feng et al. [57] regarding *Pinus yunnanensis* Franch. was found, where northwestward centroid migration under high emissions was similarly projected. However, a marked contrast was noted with Grace et al. [58] on European temperate trees, where vertical migration was identified as the primary adaptation strategy. This divergence was potentially caused by the synergy of geographic barriers and dispersal mechanisms: the N-S orientation of the Hengduan Mountains combined with avian dispersal strategies were recognized as promoting horizontal migration [59,60], whereas the E-W orientation of European mountains (e.g., the Alps) and limited dispersal capacity of wind-pollinated trees (e.g., *Picea abies* [L.] Karst) were found to necessitate vertical migration [61,62]. Critically, unrestricted migration was assumed in this model, although actual dispersal rates may lag climate velocity. This limitation underscores the necessity for future studies to be coupled with dynamic population models (e.g., Individual-Based Models), whereby seed dispersal, seedling establishment, and competition processes are simulated to enable more realistic prediction of migration trajectories. Such models should incorporate the species’ observed frost-tolerance limit (LT50 ≈ −6 °C) [63] and leaf-level water-use efficiency (δ^13^C = −29.1‰) to refine predictions of survival in novel climates.

### 4.4. Conservation Implications and Research Limitations

The following conservation strategies are proposed for *I. yunnanensis* in this study: (1) Priority protection zones should be established in current high-suitability areas to conserve extant populations and key symbiotic organisms, with reserve designs incorporating climatic refuge functions by prioritizing topographically complex regions with minimal anthropogenic disturbance [64]. (2) Ecological corridors are to be constructed along the Hengduan Mountains across provincial boundaries, avoiding major transportation arteries and agricultural development zones; native vegetation within corridors should be restored to reduce invasive species risks [65]. (3) Ex situ conservation is to be optimized through simulated future climate regimes in translocation trials, with receptor sites selected for analogous microhabitats, while monitoring the adaptive evolution of introduced individuals to prevent genetic diversity loss [66]. (4) A dynamic monitoring system is to be implemented, establishing long-term plots in low-suitability areas under SSP5-8.5 to track medicinal compound accumulation via HPLC techniques [11], assessing climate change impacts on medicinal resource quality.

Several limitations and future directions are acknowledged: First, our variable screening accounted for fine-scale collinearity (2.5 arc-min), which is pronounced in topographically heterogeneous regions like the Hengduan Mountains. At broader scales, collinearity among bioclimatic variables may diminish, reducing the need for aggressive variable reduction. Future studies should explicitly report resolution-dependent collinearity to guide variable selection. Second, interspecific interactions (e.g., mycorrhizal symbiosis dependencies) and anthropogenic pressures (e.g., medicinal harvesting intensity) were excluded from modeling, potentially causing overestimation of future suitable areas—pollinator distribution shifts may constrain reproductive success [67], while overharvesting may directly reduce populations. Third, phenotypic plasticity and genetic adaptation were unaccounted for; future integration of ecogenomic data is required to elucidate molecular mechanisms underlying climatic responses [68], such as identifying cold-tolerance genes through transcriptome analysis [69], thereby enhancing extreme climate event prediction. Although parameter optimization improved model reliability for narrow-ranged species, the static dispersal assumption inherent in current SDMs necessitates coupling with process-based models to simulate migration lag effects and population dynamics [70]. Our 2.5′ bioclimatic variables depict general habitat yet overlook slope aspect, which critically governs microclimate and soil water in the Hengduan ranges. Subsequent studies will merge DEM-derived aspects and slope and topographic wetness indices with in situ moisture–radiation measurements to generate fine-scale topo-climatic niches, improving range forecasts and locating micro-refugia for *I. yunnanensis* under warming.

## 5. Conclusions

The MaxEnt model parameters were optimized in this study (RM = 3.0, FC = QHPT), significantly reducing overfitting risk (ΔAICc = 0) and achieving high-precision predictions (AUC = 0.968). The results indicate that the current suitable habitats of *I. yunnanensis* are concentrated around the Hengduan Mountains (total area 94.88 × 10^4^ km^2^, accounting for 9.88% of China’s land area), primarily driven by temperature seasonality (Bio4, 42.5%), mean temperature of the coldest quarter (Bio11, 19.1%), and annual precipitation (Bio12, 17.5%), with optimal ecological thresholds of Bio4 = 376–597 °C, Bio11 = 5.8–12.3 °C, and Bio12 = 855–1360 mm. Under future climate scenarios, the dynamics of suitable habitats exhibit significant path dependence: in the SSP1-2.6 scenario, the area fluctuates and contracts (retention rate of 74.5% in the 2090s), while in the SSP5-8.5 scenario, the total area continues to expand (reaching 135.31 × 10^4^ km^2^ in the 2090s), although the high-suitability area shrinks by 25.58 × 10^4^ km^2^ and the low-suitability area expansion rate reaches 36.3%. Meanwhile, the distribution centroid migrates northwestward, with a displacement of 309.73 km and an elevation gain of 1483 m under the high-emission SSP5-8.5 scenario, highlighting that climate-driven niche shifts have exceeded the species’ local adaptation thresholds. This study provides precise spatial decision-making support for the conservation of *I. yunnanensis*, recommending prioritizing the protection of existing high-suitability areas in the Hengduan Mountains and constructing ecological corridors along northwest-oriented topographic pathways to facilitate climate adaptation.

## Figures and Tables

**Table 1:** Potential suitable habitat areas of *I. yunnanensis* across different periods (units: 10^4^ km^2^).

Period	Total Suitable Area	Generally Suitable Area	Moderately Suitable Area	Highly Suitable Area
Current	94.88	38.08	21.35	35.45
2050s-SSP1-2.6	101.14	50.18	20.55	30.41
2070s-SSP1-2.6	93.73	48.08	24.45	21.19
2090s-SSP1-2.6	101.24	51.08	24.62	25.53
2050s-SSP2-4.5	113.99	60.35	25.43	28.20
2070s-SSP2-4.5	110.86	59.52	23.49	27.85
2090s-SSP2-4.5	118.18	64.88	22.62	30.69
2050s-SSP5-8.5	105.85	55.98	21.45	28.43
2070s-SSP5-8.5	124.76	71.59	25.37	27.79
2090s-SSP5-8.5	135.31	80.02	29.71	25.58

**Table 2:** Suitable habitat area changes for *I. yunnanensis* under future climate change scenarios.

Period	Area (10^4^ km^2^)			Rate of Change (%)		
	Stability	Contraction	Expansion	Stability	Contraction	Expansion
2050s-SSP1-2.6	104.85	11.81	19.73	76.88	8.66	14.47
2070s-SSP1-2.6	97.35	19.31	18.09	72.25	14.33	13.43
2090s-SSP1-2.6	103.08	13.57	21.66	74.53	9.81	15.66
2050s-SSP2-4.5	105.84	10.80	34.67	69.95	7.14	22.91
2070s-SSP2-4.5	106.96	9.69	29.74	73.06	6.62	20.32
2090s-SSP2-4.5	107.85	8.78	38.01	69.74	5.68	24.58
2050s-SSP5-8.5	104.61	12.04	25.94	73.36	8.45	18.19
2070s-SSP5-8.5	100.82	15.83	53.24	59.35	9.32	31.34
2090s-SSP5-8.5	100.62	16.03	66.38	54.98	8.76	36.27

## Data Availability

The original contributions presented in this study are included in this article. Further inquiries can be directed to the corresponding author.
